# Navigating Access to Pre-exposure Prophylaxis (PrEP): Black Men Who Have Sex With Men (BMSM) Voices Living in HIV Hotspots

**DOI:** 10.1007/s40615-025-02652-x

**Published:** 2025-09-22

**Authors:** Carlos C. Mahaffey, Rohan D. Jeremiah, Andrew M. O’Neil, Déjà N. Clement, Isabel Cava, Fracisco R. Ruiz, Randolph D. Hubach, Danelle Stevens-Watkins

**Affiliations:** 1 Department of Public Health, Purdue University, West Lafayette, IN USA; 2 Department of Human Development Nursing Science, University of Illinois Chicago, Chicago, IL USA; 3 Department of Psychiatry and Behavioral Medicine, Medical College of Wisconsin, Milwaukee, WI USA; 4 Department of Psychiatry, Boston Medical Center/ Boston University Chobanian & Avedisian School of Medicine, Boston, MA USA; 5 Department of Educational, School, & Counseling Psychology, University of Kentucky, Lexington, KY USA; 6 Department of Global Studies, Purdue University, West Lafayette, USA

**Keywords:** BMSM, Black men, PrEP, HIV prevention, Barriers, Stigma

## Abstract

Pre-exposure prophylaxis (PrEP) has contributed significantly to stabilizing HIV incidence rates across most populations. However, significant disparities in HIV incidence and PrEP uptake persist among Black men who have sex with men (BMSM). This study delved into the individual, sociocultural, and structural factors associated with PrEP access and uptake among 28 BMSM residing in designated HIV hotspots. Participants were recruited from communities identified as “high priority” by the US Department of Health and Human Services’ *Ending the HIV Epidemic in the US* initiative. Participants completed semi-structured interviews focusing on PrEP awareness, access, and barriers to uptake. Guided by the Socioecological Model, key themes were identified and organized into individual, sociocultural, and structural (systemic) factors that affect PrEP access and health behaviors. Participants described individual barriers related to misinformation, potential side effects, adherence, and low perceived risk for acquiring HIV. Participants described sociocultural and structural barriers to PrEP uptake associated with stigma and PrEP shaming, affordability, the lack of physical access to providers, or non-affirming provider interactions. Overall, misconceptions, negative provider experiences, and stigma significantly limited PrEP access for the BMSM in this study. These findings underscore the need for multi-level public health approaches that address these intersecting barriers. Such strategies must be adaptable to the unique social and geographic characteristics of the BMSM population being served to optimize PrEP access and reduce HIV disparities.

## Introduction

Recent trends show that HIV infection rates have remained stable or have decreased among most groups in the USA. However, men who have sex with men (MSM) continue to be disproportionately affected, making up 67% of all new HIV diagnoses nationally in 2021 [1]. Black men who have sex with men (BMSM) represented the highest share of new HIV diagnoses among MSM (37%) and among all persons (25%) [1]. Though there have been significant scientific advancements in preventing HIV, such as HIV pre-exposure prophylaxis (PrEP), only 38% of BMSM used PrEP, despite nearly 90% being aware of PrEP in 2023 [2].

In 2012, the US Food and Drug Administration (FDA) approved the first of several medications now available to use as PrEP, Truvada®, a once-daily pill proven highly effective for reducing the risk of getting HIV [3]. Research has shown that PrEP is effective in reducing the male-to-male sexual risk of acquiring HIV by 99% [4]. However, the uptake of PrEP remains suboptimal, especially among those who could benefit most from using it [5]. Over 309,000 BMSM are estimated as potential beneficiaries from PrEP, more than the total number of Hispanic/Latino adults, including MSM (282,000), or the total number of White adults, including MSM (303,000) [6]. Although a national study by the Centers for Disease Control and Prevention in 23 US cities found that more than three-fourths of BMSM had heard of PrEP, only 19% reported its use within the past year compared to 21% of Hispanics or Latinos and 31% of White MSM [7]. Another study of BMSM in Atlanta reported that 89% were aware of PrEP, but an even smaller percentage (10%) had ever taken PrEP [8].

Reported barriers to PrEP among BMSM include stigma, concerns with cost and side effects, and self-perceived risk [9–15]. Several studies have identified these barriers to PrEP among BMSM. However, research on PrEP among BMSM has often focused exclusively on younger men [16–20] and those residing in large, metropolitan cities such as New York, Los Angeles, Washington, D.C., Atlanta, and Chicago [19, 21–23]. Furthermore, there is limited information on examining barriers to PrEP in the context of more rural and suburban environments, which typically have persistently high HIV rates despite decades of research and access to evidence-based HIV interventions [24].

### HIV Disparities in High Priority Areas

HIV stigma, rurality, lack of access to or knowledge of HIV clinics, and socially conservative culture have been found to perpetuate HIV disparities among BMSM, especially in “high priority areas” [25, 26]. “High priority areas” as designated by the US Department of Health and Human Services (HHS) are defined as locations where more than 50% of HIV diagnoses occur. These locations include (1) the District of Columbia; San Juan, Puerto Rico; and 48 US counties and (2) seven states with a disparate amount of HIV cases occurring in rural areas (population less than 50,000) [27, 28]. In addition to the previously mentioned factors, HIV disparities (and health disparities, overall) affecting BMSM have been linked to widely studied race-based discrimination and medical distrust in the USA [29, 30]. These statistics on HIV disparities among BMSM underscore the need for novel HIV prevention efforts, particularly for BMSM residing within communities outside of large, metropolitan areas [1].

### Theoretical Framework and Purpose

The purpose of this study was to examine the perceptions of and access to PrEP among BMSM residing in HIV hotspots. We utilized the socioecological model as a theoretical framework to guide this study. The socioecological model has been used as a framework to examine PrEP barriers among BMSM, identifying how each factor is associated with conditions of specific socioecological levels [15].

## Methods

This study is part of a larger study examining substance use, sexual risk behavior, and healthcare utilization among BMSM. While the goal of the larger study was to identify factors contributing to health inequities among BMSM, including HIV, we found that a gap remained in qualitatively examining participants’ perceptions of and access to PrEP. We were especially interested in understanding how access to PrEP may be inhibited across individual, sociocultural, and structural levels. All protocols for research with human subjects were approved by the institutional review board (protocol #42758).

### Participant Recruitment and Data Collection

Recruitment efforts consisted of distributing flyers to four local, non-profit organizations in Lexington, KY, and Oklahoma City, OK, with foci of delivering HIV prevention/treatment programs and services to residents, specifically BMSM. Flyers included the study aims, explanation of what consenting participants would be asked to do, eligibility requirements, and compensation for participating in the study. Flyers were also placed in local LGBTQ+ bars and clubs. Additionally, flyers were shared in private Facebook groups of local, Black LGBTQ+ persons. Members of those private Facebook groups were also asked to share the flyer with their network [31–33].

Recruitment for this study began in Lexington, KY. Established research partnerships with non-profit organizations (from other studies) within the Lexington community were leveraged to meet and develop a relationship with a local leader from the Black LGBTQ+ community. From this relationship, we were able to identify potential recruitment spaces and networks, which led to participants in Louisville, KY, and other states, including Ohio, Georgia, and Oklahoma. Overall, participants were recruited from Georgia (Atlanta), Kentucky (Lexington and Louisville), Ohio (Cincinnati), and Oklahoma (Oklahoma City). Each of these areas is described as a HIV hotspot for infection and thus designated as a priority jurisdiction of *Ending the HIV Epidemic in the US*, the US federal response to addressing the HIV epidemic [28].

Participants were eligible for this study if they were assigned male sex at birth, were proficient English speakers, aged 18 or older, self-identified as Black or African American, and acknowledged having anal or oral sex with another male in the past year. We used an online screening questionnaire to determine eligibility. Eligible men who wished to participate in the study were invited for an in-person interview at a local community-based organization or another suitable space that allowed for privacy and confidential discussions. For each participant, the informed consent was read aloud by a trained interviewer from the research team as the individual followed along, then signed before beginning the interview.

Qualitative data were collected from 28 BMSM through one-on-one, semi-structured interviews with a research team member. Interviews were conducted in public libraries, community centers, and private, off-campus office spaces according to the participant’s expressed need for confidentiality. Study participant interviews lasted between 45 and 75 min, with most lasting an hour. Individuals who completed the interview were compensated $25 cash for their study participation.

Data collection continued until topic saturation (when further data gathering adds little new information to topic conceptualizations) was reached [34]. For this study, saturation occurred during the data analysis from the 25th interview. Reviews of previous studies on the sexual health of MSM that have used qualitative methodology indicate that sample sizes of 20 or more participants were common [35–37]. Recruitment and interviews took place between March 2017 and September 2018.

### Study Instruments

A study interview guide was developed by the first author and piloted among persons meeting the study criteria and other researchers who self-identified as Black and members of the LGBTQ+ population. Feedback was provided by each and incorporated into the guide before the first study interview. The interview guide included questions about sociodemographic information, awareness of HIV pre-exposure prophylaxis (PrEP), assessing knowledge of PrEP, and factors that influence persons’ perceptions and motivation to take or not take PrEP. These content-specific questions were adapted from previous research studies [38–41]. Other questions were developed to gain a better understanding of participants’ perceptions and potential barriers towards PrEP. Sample questions included “Before today, had you previously heard about PrEP?”; “What types of things have you heard, or might you be concerned about, regarding using PrEP as prevention for HIV?”; “What do you feel are the negative ideas about taking PrEP?”; “How, if at all, does the availability of PrEP and other HIV treatment affect your ideas about using condoms during sex?”

### Positionality

The research team consisted of a Black, cisgender, bisexual male who identifies as part of the research population, BMSM; a Black, cisgender, heterosexual female with experience conducting research with Black persons who identify as LGBTQ+; and a White, cisgender, gay male, with experience coding qualitative interview transcripts of studies involving rural, LGBTQ+ populations. Although the team represented diverse backgrounds and experiences that aligned with the study's population of interest, we were cognizant of the study’s power dynamic during the recruitment, data collection, and analysis phases. To minimize such biases within this study, the research team proactively engaged in numerous reflexivity, discussion, and debriefing points to refine the study codebook and analysis [42, 43]. Specifically, the research team individually analyzed data then used rater triangulation to ensure that themes were accurately identified from the data and bias accounted for from each researcher’s shared experience and inexperience with the study population.

### Data Analysis

Descriptive analyses were conducted using SPSS statistical software (version 26) to report the sample sociodemographics. The Qualitative Pathway Project Mapping Tool (QL-PPMT) was used to ensure methodological rigor [44]. Primarily, the QL-PPMT was used in this study to guide the data analysis as well as to ensure that the procedures conducted in this qualitative study were planned, tracked, and discussed thoroughly. The QL-PPMT is geared towards new investigators, students, and trainees and is used to provide the core components needed for developing rigorous qualitative research methods. The aim of this tool is to inform the development of qualitative research proposals that meet the criteria set by the NIH.

The interviews were digitally audio-recorded, professionally transcribed verbatim, and double-checked for accuracy against the recordings. After collecting the qualitative data, the study team transcribed and double-checked it for accuracy. Based on the interview guide, a code book using a priori categories and codes was developed. Content analysis was performed by inductively identifying and interpreting concepts and themes from the interview transcripts [34]. This method involved multiple readings of transcripts and interview notes and analytic induction via open and axial data coding using Dedoose (version 8.0.35) to organize transcripts. The team utilized a hybrid thematic approach where certain themes were deductively decided based on theory and the interview guide, while others were established inductively based on the information extracted from the collected data.

Coding was checked by the primary investigator, and discrepancies were resolved through intercoder reliability checks. The team refined the codebook throughout the process as connections were drawn between ideas. Findings were organized around domains of inquiry.

## Results

### Participant Sociodemographics

In total, 28 interviews were conducted. Participants’ age ranged from 21 to 57 years (*M* = 32.9, SD = 9.24). Overall, participants were highly educated; nearly 90% have postsecondary education, with 10 participants receiving some college and nine earning a bachelor’s degree. Six participants earned a master’s degree. Most of the participants were single (68%) and employed (86%). Of those with health insurance (*n* = 25), 64% had private health insurance compared to 36% of participants who had Medicaid. More than 46% of the sample reported a yearly personal income of less than $30,000. A quarter of the sample reported a yearly personal income of less than $10,000 (see Table 1).

**Table 1:** Sociodemographic characteristics of the sample (*N* = 28)

	*N*	%	*M*	SD
Age			32.86	9.24
Location (state)				
Georgia	3	10.7		
Kentucky	18	64.3		
Ohio	2	7.1		
Oklahoma	5	17.9		
Education				
High school graduate	3	10.7		
Some college or technical school	10	35.8		
Undergraduate degree	9	32.1		
Graduate degree	6	21.5		
Personal income				
Less than $30,000	13	46.4		
$30,000 to $49,999	8	28.6		
$50,000 to $69,999	5	17.8		
More than $70,000	2	7.1		
Employment status				
Full-time (35+ hours per week)	20	71.4		
Part-time	4	14.3		
Retired/disability	2	7.1		
Not in workforce	2	7.1		
Insurance status				
Employer health insurance	16	57.1		
Medicaid	9	32.1		
Not insured	3	10.7		
Relationship status				
Single	19	67.9		
Married or exclusive partnership	9	32.1		
Ever heard of PrEP				
Yes	24	85.7		
No	4	14.3		
Prescribed PrEP				
Yes	6	21.4		
No*	22	78.6		

^*^Note: includes three participants who are HIV +

### PrEP Awareness

The overwhelming majority of participants (86%) said they had heard of PrEP before the interview. The most common sources of PrEP information were friends, a health provider, social media (including hook-up apps), and news articles. Despite this knowledge, 79% of participants had never taken PrEP.

### PrEP Barriers

Barriers to PrEP access were associated with three distinct socioecological levels: (1) individual factors, (2) sociocultural factors, and (3) structural factors. To contextualize these barriers, the findings are presented in Table 2 using themes and verbatim quotes from BMSM in this study. Each participant was assigned a pseudonym in place of their name to protect privacy and confidentiality. The implications of these findings and how they overlap are discussed later.

**Table 2:** Barriers and selected quotes

Factors (*n*)	Selected quotes
**Individual—**Individual level concerns regarding PrEP typically raised by BMSM, including lack of knowledge or misinformation (12), concerns about short and long-term effects (12), adherence (11), and low perceived risk of acquiring HIV (13)	It’s [PrEP] been talked about a little bit more, yeah, because mostly you see just white guys taking it. You don’t see a lot of Black people taking it and I think that’s just because of the limited access to healthcare. They [BMSM] just don’t know. **(Barry, 50, KY)** I feel if antibiotics can [lead to altering normal body functions in negative ways] – if they tell you that you should only be on antibiotics for so many days because it can cause this issue, I’m sure an anti-viral of that magnitude could cause you some type of health issue for being on it on a very regular basis. **(Aiyden, 26, KY)** I guess I wouldn’t [take PrEP] – Unless my wife is having other type of sex with people without condoms. You know what I mean? It’s like –I wouldn’t think I need it. **(Amari, 40, KY)**
**Sociocultural—**Stigma and PrEP shaming (15)	I think there’s a perception that those guys – guys on PrEP – are more sexually active you know so they’re more promiscuous and so there’s that stigma around promiscuity and being fast or however you want to say it. I think that’s tied to guys on PrEP because it’s like, ‘Well, you wouldn’t need PrEP if you weren’t overly sexually active.’ **(Ian, 24, OK)** Because just like me, how I thought PrEP was associated with people with HIV, I think there are still a lot of people still out there, kind of still thinking the same thing. So, when you go out and have a conversation like I’m on PrEP, people might automatically assume. If I asked you, do you have HIV, but people see PrEP might automatically assume that you’re HIV positive. **(Zaire, 25, KY)** It was a small town. They talk. I feel like the confidentiality breach was broken only because I stepped foot into the health department. **(Otis, 28, OK)** One time this guy – this Black doctor – was like, ‘Okay, you know you need to slow down. You were just in here about this.’ He didn’t even know because I was having discharge coming out of my urethra or whatever, but it wasn’t an STD or anything. I thought it was abnormal or something like that and he assumed that it was that [an STD] or whatever and he tried to give me this big speech about it. **(Brenton, 25, OK)**
**Structural (systemic)—**Concerns about affordability (10), access to PrEP and where to receive additional PrEP education (5); interacting with providers (9); disclosing sexual information with providers (11); medical mistrust and provider preferences (11)	It’s difficult to find providers who will [prescribe PrEP] – in fact, at one point in time there was only one provider who would do it on a regular basis. So, there was no PrEP clinic. For many years, a lot of doctors here weren’t familiar with it. They felt like, ‘I’m not gonna give you an HIV drug when you don’t have HIV. **(Demarcus, 37, KY)** [I don’t know] how to get it [PrEP] [and] I probably couldn’t afford the prescription. **(Marques, 26, KY)**

*n*,  the number of participants who endorsed each theme

### Individual Factors

#### PrEP Knowledge and Assumptions

While most of the participants had heard of PrEP, the accuracy of their PrEP knowledge varied greatly. Four participants described PrEP and PrEP users as being HIV-positive and taking medications for treatment as opposed to some of the same drugs being prescribed to HIV-negative persons for HIV prevention. Additionally, two participants reported skepticism of the priorities of healthcare and the pharmaceutical industry regarding PrEP. For example, upon learning about the effectiveness of PrEP in reducing the risk of HIV transmission, Dwyane (49 years old, KY) stated that it was “weird how they can give a pill [for PrEP] to keep from getting it [HIV], and not a pill to cure it [HIV].”

Participants also demonstrated an overall lack of knowledge about PrEP. Notably, five participants reported only recently learning about PrEP from seeing it in profiles on LGBTQ+ social networking, dating, or hook-up apps (e.g., Grindr®). One participant (Jamar, 29 years old, OK) was unclear of who PrEP was for; he said to the interviewer, “I don’t know if you know or anything like that, because I think I tried to ask – like, if you’re undetectable, would you be able to still take PrEP?”. Barry (50 years old, KY) said, “It [PrEP] has been talked about a little bit more, yeah, because mostly you see just White guys taking it. You don’t [see] a lot of Black people taking it, and I think that’s because of the limited access to healthcare. They just don’t know.”

#### Medication Concerns

Many individually held barriers were related to medication concerns. Specifically, 12 participants had concerns about the potential side effects of taking PrEP.

Barry (50 years old, OK) said, “I probably wouldn’t take it because I heard it gives you osteoporosis. Your bone density mass drops. That’s what I’ve learned that’s stopping a lot of Black men from taking it.”

Similarly, two participants described concerns of potential side effects from long-term use leading to permanent damage to organs. These concerns could negatively affect willingness to initiate PrEP and adherence to PrEP.

#### Adherence Concerns

Participants discussed concerns about PrEP adherence. Specifically, 11 participants expressed that if they did take PrEP, they would have trouble remembering to take a pill every day. Deion (44 years old, KY) acknowledged that remembering to take a daily pill could be challenging and expressed a willingness to take PrEP if it were available in injection form. Deion stated “That [injectable PrEP] would assure me that I’m okay, I just have that one appointment, three months, [and] come back.”

#### Perceived Risk

A common individual-level barrier was having a low perceived risk of acquiring HIV. Notably, 13 participants described not feeling the type and frequency of sexual activity they engaged in would warrant taking PrEP daily, despite meeting PrEP guidelines. When asked about reasons not to take PrEP, Amari (40 years old, KY) described not needing PrEP unless his wife was having sex with other people without condoms, completely disregarding the risk he assumes for himself (and his wife) by having unprotected sex with multiple partners. Khalil (23 years old, OK) described having a “slim to none” chance of getting HIV. However, when asked about his condom use and sexual risk behavior, Khalil stated:

I am the kind of person like I am super adventurous. I love to have fun, try anything once, and so I know that sometimes I can get the best of myself maybe you could say and just make a dumb decision just because. And I mean I don’t know, it might pertain more to them being like super hot. I don’t know.

### Sociocultural Factors

#### Stigma and PrEP Shaming in the LGBTQ+ Community

Participants described stigma as a sociocultural barrier. Notably, two men expressed a reluctance to use PrEP due to a common misconception held by others that persons on PrEP are HIV positive and using it for treatment, not HIV prevention. For example, Zaire (25 years old, KY) described how others may view a person taking PrEP as “automatically” being HIV positive. He expressed having agreed with this view in the past. Demarcus (37 years old, KY) also stated, “if I had [PrEP] pills in my bag and a dude comes over, he doesn’t know if it’s safe. I mean, he doesn’t know if it’s HIV medicine or PREP.” Two other participants indicated they were aware of other men reporting taking PrEP (which is specifically labeled for HIV prevention) as a means not to disclose their HIV-positive status. As a result, those participants identified PrEP as HIV treatment.

Participants also discussed PrEP-shaming due to the association of its use with promiscuity in the gay community. an (24 years old, OK) explained:

It’s like, ‘Well, you wouldn’t need PrEP if you weren’t overly sexually active. You wouldn’t need PrEP if you stay with one partner.’ So, there’s that stigma around sexual activity and too much sexual activity and promiscuity along with taking PrEP. So, I think even internally there’s an association of shame because I think there is a shame – a subtle shame – that still lies with me and promiscuous activity that I previously have had and taking PrEP.

#### Provider-Based Stigma

Brenton (25 years old, OK) described having several negative interactions with providers, in which the doctors made assumptions based on stigma “because they’re homophobic”:

One time this guy – this Black doctor – was like, “Okay, you know you need to slow down. You were just in here about this.” He didn’t know because I was having discharge coming out of my urethra or whatever, but it wasn’t an STD or anything. I thought it was abnormal or something like that, and he assumed that it was that [an STD] or whatever, and he tried to give me this big speech about it.

### Structural (Systemic) Factors

#### Affordability

Participants gave very brief remarks regarding the cost of PrEP. For example, lack of insurance coverage was a barrier to PrEP access, with four participants explicitly describing “not having insurance” as their primary reason for not taking PrEP. Six participants with private insurance also highlighted cost as a consideration to use PrEP. Cameron (37 years old, OH) reported that he was considering PrEP but would be “concerned about it [PrEP]” if it was not covered by his insurance.

#### Lack of Access

Another barrier to PrEP described by two participants was the lack of access to healthcare providers and clinics. Demarcus (37 years old, KY) described himself as an activist for LGBTQ + health issues and stated:

It’s been difficult as someone who is trying to get other people to go on it [PrEP] and tell other people about it. It’s difficult to find providers who will – in fact, at one point in time there was only one provider who would do it on a regular basis. So, there was no PrEP clinic.

Furthermore, three participants did not know where to start in wanting to access PrEP. Marques (26 years old, KY) stated that he “didn’t know how to get it [PrEP].”

#### Provider Interaction

Eight respondents also cited medical providers as a structural barrier, saying that they were either unwilling to prescribe PrEP or were not affirming their sexual behaviors. Demarcus (37 years old, KY) described his experience: “For many years, a lot of doctors here weren’t aware of it. They felt like, ‘I’m not gonna give you an HIV drug when you don’t HIV.’ They saw it as a – they understood PEP [Post-exposure Prophylaxis].” Another participant (Lavaughn, 29 years old, KY) described an experience with a provider who determined he was not a good candidate for PrEP. The participant expressed having some regret after the interaction with the provider because they felt “there were more benefits than risks” with taking PrEP.

#### Medical Mistrust and Provider Preference

Medical mistrust was expressed by three participants regarding the confidentiality of medical information. Otis (28 years old, OK) described a challenging experience “growing up in small town USA” as “it really affected me just because – even to go in [health department] and get an HIV test – it was like everyone knows what’s happening like, ‘I can’t do this.’” Separately, eight participants discussed holding a provider preference. Participants described having more trust and comfort with a health provider that shared their culture. For example, Darnell (30 years old, KY) stated that he “wanted [a Black provider] because the Caucasian race don’t experience the same things that we do when it comes to health issues and something like that.”

## Discussion

This study aimed to examine how access to PrEP may be inhibited across individual, sociocultural, and structural levels among BMSM. There are three main points that emerged from the findings that warrant further discussion: (1) men hold multiple misconceptions about PrEP, (2) limited options and negative experiences with providers limit the ability of BMSM to access PrEP, and (3) stigma and PrEP shaming exist across multiple levels, impacting PrEP uptake.

As initial studies showed that PrEP awareness was greater among those who were White, older, more educated, had a high socioeconomic status, and were more open about their sexual orientation [45, 46], efforts were made to improve PrEP awareness. Although the promotion and dissemination of PrEP information through large-scale public health campaigns improved awareness, PrEP misconceptions and overall knowledge about the medication persist as significant challenges [10, 47]. In this study, misconceptions about PrEP held by BMSM included overestimating negative outcomes from potential side effects [48]. These misconceptions, along with other medication concerns, have been identified as primary barriers to initiating PrEP [9, 49, 50].

In this study, BMSM highlighted challenges in locating PrEP-knowledgeable healthcare providers and reported negative experiences when discussing PrEP and their sexual health in general. Aside from a few participants in large, urban areas, many participants in this study living in more rural and suburban environments were unsure where to access PrEP or PrEP providers. The inability to access PrEP providers, irrespective of PrEP awareness, has been recognized as a significant barrier to PrEP uptake [10]. Even BMSM who use PrEP and were unable to communicate with providers have reported concerns about adherence and subsequently discontinued PrEP [51].

Many participants who had access to healthcare providers reported non-affirming experiences. They described providers speaking in a condescending manner and believing that their confidentiality would be compromised by healthcare staff or the provider themselves. Similar research has indicated these experiences are exacerbated by providers harboring negative attitudes, a deficiency in PrEP knowledge, and a lack of comfort in prescribing PrEP [52–55]. Some BMSM in this study believed that having a Black provider would be more relatable and culturally affirming. Research indicates that BMSM perceive Black providers as more relatable, capable of providing higher-quality care, and exhibiting less homonegativity, which in turn could increase their willingness to use PrEP [30, 56, 57]. However, a notable finding from the current study revealed a participant’s negative experience with a Black, male provider, who was described as exhibiting homophobic behavior. These opposing experiences highlight the complexity of patient-provider dynamics and suggest that provider characteristics beyond race may influence BMSM perceptions and healthcare experiences. Therefore, future research is needed to explore this topic further.

Stigma was a significant barrier to PrEP among most participants, manifesting across sociocultural and structural levels. Specifically, participants described apprehension about using PrEP due to negative attitudes and perceptions from their social networks, including friends, potential sexual partners, and other members of the LGBTQ+ community. Participants also described experiencing stigma from healthcare providers. They explained that some providers associated PrEP users with promiscuity, leading to feelings of judgment. These findings align with previous research highlighting the association between increased stigma surrounding PrEP and perceptions of promiscuity, particularly among BMSM [18, 58, 59].

It is important to consider that the majority of participants in this study reside in more suburban and rural areas. While the findings of this study align with those of previous studies conducted among BMSM in large metropolitan areas, certain aspects of these settings may intensify the barriers to PrEP access we have identified [52, 60–64]. For instance, stigma has been identified as a more significant obstacle to PrEP uptake in Southern states [60] and other cities and towns located outside of large metropolitan areas [61]. Furthermore, medical mistrust has been found to be more deeply entrenched among BMSM in Southern states compared to other MSM [52]. These contextual factors emphasize the need for tailored approaches to address PrEP access in diverse geographic and social landscapes [65].

### Limitations

Although this study confirms and provides greater nuance to previous research, some limitations should be noted. First, most participants were recruited through convenience sampling using HIV-focused community organizations to identify eligible participants. This could contribute to the high percentage of PrEP awareness among the study sample. Second, most of the sample is from one state, Kentucky. While the objective of the study was to understand common themes among BMSM residing in HIV hotspots, the small number of participants from other states restricts the ability to report that the findings from this study are representative of the larger population of BMSM residing in those HIV hotspots across other Southern states. Third, this study relied on self-reported knowledge, perceptions, and experiences with an interviewer, which could have led to biased responses to the interview questions. Finally, the high percentage of participants with post-secondary education is not representative of the US or Black American population, affecting this study’s transferability.

### Implications and Future Studies

The findings from this study align with previous research and advance our understanding of how various factors influence the ability and willingness of BMSM to access PrEP in HIV hotspots. Future research is necessary to evaluate strategies for addressing stigma, improving providers’ PrEP knowledge and cultural competency, and informing PrEP uptake. These programs must be adaptable to the unique characteristics of the BMSM population being served in diverse geographical areas, with a particular focus on HIV hotspots in the Southern USA. This targeted approach is essential for optimizing PrEP access and uptake, and ultimately, reducing HIV disparities among BMSM.

## Conclusion

Considering ongoing federal and state-level challenges to LGBTQ+ and Diversity, Equity, and Inclusion (DEI)-focused rights and research initiatives, understanding and addressing barriers to PrEP uptake among BMSM has become critically important. The current policy landscape underscores the urgency of this work to mitigate potential negative impacts on public health, health equity, and the fight to end the HIV epidemic. Therefore, a multi-pronged approach is essential to address the barriers to PrEP expressed by BMSM. This approach must address issues regarding PrEP knowledge and misinformation, provider-level knowledge and interaction with BMSM, and stigma, while also considering the unique social and geographic characteristics of the environments where BMSM reside.

## Data Availability

Due to the sensitive and identifiable nature of the qualitative interviews, the data supporting this study’s findings are not publicly available. This is in accordance with ethical approval and participant consent.
